# Comparative efficacy of second- and third-generation fluoroquinolone antibiotics against systemic Escherichia coli infection in broiler chickens in Qalyubia Governorate, Egypt

**DOI:** 10.1038/s41598-026-59593-6

**Published:** 2026-07-01

**Authors:** Ali M. Asy, Amany O. Selim, Dalia M. Azab, Eman A. Abozeid, Mona A. Abd-Elrehim, Marionette Z. Nassif, Gehan El-Saied

**Affiliations:** 1 Biochemistry Department (Pharmacology), Animal Health Research Institute, Benha Branch, Agriculture Research Center (ARC), Giza, Egypt; 2 Bacteriology Department, Animal Health Research Institute, Benha Branch, Agriculture Research Center (ARC), Giza, Egypt; 3 Biochemistry Department, Animal Health Research Institute, Benha Branch, Agriculture Research Center (ARC), Giza, Egypt; 4 Food Hygiene Department, Animal Health Research Institute, Benha Branch, Agriculture Research Center (ARC), Giza, Egypt

**Keywords:** Levofloxacin, Enrofloxacin, *Escherichia coli*, Antioxidant enzymes, Broilers, Diseases, Microbiology

## Abstract

Avian pathogenic *Escherichia coli* (APEC) causes severe colibacillosis in poultry. This study compared the efficacy of the third-generation fluoroquinolone levofloxacin and the second-generation enrofloxacin against APEC. In vitro, 155 samples (livers, hearts, and lungs) from broiler farms in Qalyubia, Egypt, yielded 43 *E. coli* isolates, of which 16 formed strong biofilms. Antibacterial sensitivity testing revealed that levofloxacin achieved a higher susceptibility rate (43.75%) and larger inhibition zones (26 mm) compared with enrofloxacin (18.75%, 13 mm), indicating superior antibacterial activity. In vivo, 150 broiler chicks were experimentally infected with APEC O2 and allocated to five groups: negative control, positive control, levofloxacin-treated, enrofloxacin-treated, and enrofloxacin-colistin combination. Levofloxacin treatment resulted in significantly higher cumulative body weight gain and lower mortality (2460.37, 3.33%), compared with enrofloxacin (2126.09, 6.67%) (*P* < 0.05). Levofloxacin was further associated with improved hematological and biochemical profiles, reduced oxidative stress markers (MDA, SOD, GSH), and lower bacterial counts in visceral organs (*P* < 0.05). Collectively, these findings indicate that levofloxacin offers enhanced therapeutic efficacy over enrofloxacin in the treatment of avian colibacillosis under the conditions of this study.

## Introduction

Colibacillosis, primarily caused by avian pathogenic *Escherichia coli* (APEC), is a significant bacterial disease in broiler chickens that drives high mortality rates and substantial economic losses in the Middle East and the global poultry industry. The infection can disseminate to internal organs, causing septicemia, peritonitis, and air sacculitis^1,2^. Beyond its impact on animal health, APEC, a member of the family Enterobacteriaceae (now reclassified within Pseudomonadota), poses a potential zoonotic risk and is frequently employed as an indicator organism for enteric pathogen contamination^3^. Certain APEC strains share notable genetic similarities with human extraintestinal pathogenic *E. coli*, including uropathogenic *E. coli* and neonatal meningitis *E. coli*. This suggests that APEC may have the capacity to cause urinary tract infections and meningitis in humans^4^. APEC’s capacity to disseminate antimicrobial resistance (AMR) genes via horizontal gene transfer represents a serious threat to both animal and public health. Effective control demands enhanced biosecurity measures in poultry operations, judicious antibiotic stewardship, and continuous surveillance of resistant strains^5^. Fluoroquinolones (FQ) are synthetic bactericidal agents sharing a bicyclic core related to 4-quinolone; they are widely used in human and veterinary medicine for a broad range of serious bacterial infections, particularly against *Escherichia coli* and other Gram-negative bacteria^6^. In poultry, FQ have been extensively employed to control avian colibacillosis^7^. Their bactericidal activity derives from inhibition of topoisomerase enzymes—specifically DNA gyrase (topoisomerase II) in Gram-negative bacteria and topoisomerase IV in Gram-positive organisms- disrupting DNA replication and transcription and ultimately leading to bacterial cell death^8^. Enrofloxacin, a second-generation fluoroquinolone, has long been used in veterinary medicine for colibacillosis; however, its use has come under increasing scrutiny owing to rising AMR and regulatory restrictions imposed by authorities such as the European Medicines Agency (EMA)^9^. Consequently, there is an urgent need to identify alternative therapeutic agents capable of effectively managing colibacillosis while minimizing resistance development. Levofloxacin, the L-enantiomer of ofloxacin and the third-generation fluoroquinolone, exhibits a broader spectrum of activity and an improved pharmacokinetic profile, including near-complete oral bioavailability^10^, making it a promising candidate for infections caused by APEC. This study aimed to evaluate the in vitro susceptibility of *E. coli* isolates from broiler farms in Qalyubia, Egypt, to enrofloxacin and levofloxacin, and to determine the prevalence of fluoroquinolone-resistant *strains*. In addition, a comparative in vivo assessment of the clinical, hematological, biochemical, oxidative-stress, and microbiological outcomes of both agents was conducted in broilers experimentally infected with APEC, with the goal of informing optimized therapeutic protocols and antimicrobial stewardship in veterinary medicine.

## Materials and methods

### In vitro study

#### Sample collection

We collected 155 samples (liver, heart, and lungs) from freshly dead and septicemic broiler chickens older than two weeks showing symptoms and postmortem lesions of colibacillosis. Samples were obtained from broiler farms across various areas of Qalyoubia Governorate, Egypt, between 2024 and 2025. No consistent records of prior antibiotic use were available from the sampled farms. Written permission was obtained from farm owners prior to sampling. Samples were collected in sterile tubes containing buffered peptone water and then transferred immediately to the laboratories of the Animal Health Research Institute (AHRI), Benha Branch.

#### Isolation and identification of *E. coli*

Collected samples were initially pre-enriched in nutrient broth at 37 °C for 24 h. E. coli isolation was performed on selective media: Eosin Methylene Blue (EMB) agar (OXOID) incubated at 37 °C for 24 h, and Tryptone Bile X-glucuronide (TBX) agar (Hi Media) incubated at 44 °C for 24 h. Characteristic colonies (metallic green on EMB and blue-green on TBX) were purified on tryptic soy agar for biochemical testing. Pure isolates were then preserved in brain heart infusion broth supplemented with 30% glycerol at − 20 °C for subsequent analysis, following Markey et al.^11^ and ISO 16649-2 guidelines.

#### Serological identification of *E. coli*

Isolates were serotyped at AHRI, using “SEIKEN” polyvalent and monovalent O and K antisera (MAST ASSURE™), according to the method of Markey et al.^11^. Briefly, a loopful of a 24-hour-old colony grown on nutrient agar was emulsified in physiological saline on a glass slide, mixed with a drop of antiserum, and observed for agglutination over one minute.

#### Antibacterial sensitivity testing

Disk diffusion susceptibility testing was performed according to CLSI 2024 guidelines. Antimicrobial discs tested included levofloxacin, enrofloxacin, norfloxacin, colistin, and levofloxacin + nosihertide were used. Nosihertide was tested in combination with levofloxacin to assess potential synergistic antimicrobial effects.

#### Biofilm formation and antibiotic penetration assay

biofilm-forming capacity of the *E. coli* isolates was assessed using Congo Red Agar (CRA). Isolates forming black colonies with a dry, crystalline morphology were classified as strong biofilm producers; those remaining pink were classified as weak producers. The assay was performed in triplicate on three independent occasions, with *Edwardsiella tarda* MW362141 included as a positive control, following Pramodhini et al.^12^. Biofilm formation was also induced in 96-well microtiter plates. After 24 h, antibiotics were applied to established biofilms, and residual viable cells were quantified by plating on MacConkey agar and enumerating colony-forming units (CFU). The percentage reduction in CFU counts post-treatment served as an index of biofilm penetration efficacy for each antibiotic.

### In vivo study

#### Birds

A total of 150 apparently healthy, Day-Old Chicks (DOC), unsexed broilers of the Cobb 500 commercial breed, were obtained from El-Watania Poultry Company.

#### Drugs

Levofloxacin (Lexoflon OR^®^, NITA-FARM, Russia. 150 mg/ml). Enrofloxacin (Enroveto 20^®^, VMD, Belgium. 200 mg/ml). Enrofloxacin + Colistin (Enronit OR^®^, NITA-FARM, Russia. 100 mg enrofloxacin + 1000 000 IU colistin per ml).

#### Experimental design

One hundred and fifty chicks were randomly allocated to five groups of 30 chicks each. Group 1 (negative control) comprised non-infected, non-treated birds. Groups 2–5 were orally challenged on day 14 with APEC serotype O2, one of the most prevalent strains in the Middle East and North Africa (MENA) region. The challenge dose was 0.5 ml of bacterial suspension (9.0 × 10⁸ CFU/ml) per bird. Group 2 (positive control) was infected but untreated. Group 3 was treated with Levofloxacin at 7.5 mg/kg body weight (BW) in drinking water for five consecutive days (days 16–20). Group 4 was treated with enrofloxacin at 10 mg/kg BW in drinking water for five consecutive days (days 16–20). Group 5 received enrofloxacin (10 mg/kg BW) combined with colistin (500,000 IU/kg BW) in drinking water for five consecutive days (days 16–20). All birds were offered a commercial, antimicrobial-free balanced ration and water ad libitum throughout the experiment.

#### Growth performance parameters

Chicks were individually marked and weighed at one day old, then weekly through week 5. Cumulative feed consumption, cumulative body weight gains (BWG), and feed conversion ratio (FCR) were calculated^13^. Mortality was recorded daily throughout the experimental period.

#### Hematological parameters

Blood samples were collected from the brachial vein of five birds per group on days 21, 28, and 35. Each sample was divided into two aliquots: one collected into EDTA-containing tubes for determination of erythrocyte count (RBC), total leukocyte count (WBC), hemoglobin (Hb) concentration, and packed cell volume (PCV)^14^; the second was allowed to clot, and serum was separated and stored at − 20 °C for biochemical analysis^15^.

#### Biochemical parameters

Serum samples were analyzed for alanine aminotransferase (ALT) activity^16^, Aspartate aminotransferase (AST) activity^16^, uric acid concentration^17^., and creatinine concentration^18^.

#### Oxidative stress parameters

Liver and kidney tissues were obtained from five birds per group on days 21, 28, and 35 and stored at -20 °C. Tissue homogenates (10% w/v in normal saline) were prepared for determination of malondialdehyde (MDA) concentration^19^, superoxide Dismutase (SOD) activity^20^, and reduced glutathione (GSH) concentration^21^.

#### Microbiological efficacy

APEC was isolated from five samples per group, collected from bone marrow, liver, kidney, spleen, and lungs at days 13, 15, and 21 of age, following Quinn et al.^22^.

#### Statistical analysis

Data normality was assessed using the Shapiro–Wilk test. Between-group differences were analyzed by one-way ANOVA followed by Duncan’s multiple range post hoc test^23^. Statistical analyses were performed using IBM SPSS Statistics v28.0 (SPSS Inc., Chicago, IL, USA). Results are expressed as mean ± standard error of the mean (SEM), and *P* < 0.05 was considered statistically significant^24^.

#### Ethical approval and ARRIVE guidelines

All animal procedures were approved by the Research Ethics Committee of the Animal Health Research Institute, Agriculture Research Center (ARC), Giza, Egypt (Approval No.ARC AHRI 16/26). All methods involving animals are reported in accordance with the ARRIVE guidelines (https://arriveguidelines.org/) to ensure transparent and comprehensive reporting.

## Results

### In vitro studies

#### Isolation and identification of *E. coli*

Of the 155 samples, 43 isolates were confirmed as *E. coli*. 34 from liver, five from lung, and four from heart.

#### Serological identification and biofilm characterization

Among the 43 isolates, 16 were classified as strong biofilm producers and subjected to serotyping (Table 1). The predominant serovar was O2:K1 (37.50%), followed by O44:K74 (25.00%), O26:K60 (18.75%), O55:K59 (12.50%), and O114:K90 (6.25%).

**Table 1:** Serotypes of strong biofilm-forming *E. coli* isolates.

E. coli isolate	No. of isolates	Percentage (%)
*E.coli* O2:K1	6	37.50
*E.coli* O44:K74	4	25.00
*E.coli* O26:K60	3	18.75
*E.coli* O55:K59	2	12.5
*E.coli* O114:K90	1	06.25
Total	16	100.00

#### Antibacterial sensitivity testing

In vitro sensitivity testing of the 16 strong biofilm-forming isolates demonstrated superior efficacy of levofloxacin over enrofloxacin (Table 2). Levofloxacin achieved the highest susceptibility rate at 43.75% (7/16 isolates) compared with 18.75% (3/16 isolates) for enrofloxacin. The levofloxacin-nosihertide combination yielded 50.00% sensitivity (8/16 isolates). Resistance was most prevalent against enrofloxacin (50.00%; 8 isolates), followed by levofloxacin (37.50%, 6 isolates). Levofloxacin produced the largest mean inhibition zone (26 mm), significantly exceeding those of enrofloxacin (13 mm) and colistin (12 mm).

**Table 2:** Antibacterial susceptibility profile of strong biofilm-forming *E. coli* isolates.

Category	Levofloxacin		Levofloxacin + Nosihertide		Enrofloxacin		Colistin	
	#	%	#	%	#	%	#	%
Sensitive	7	43.75	8	50.00	3	18.75	9	56.25
Intermediate	3	18.75	3	18.75	5	31.25	3	18.75
Resistant	6	37.50	5	31.25	8	50.00	4	25.00
Total	16	100.00	16	100.00	16	100.00	16	100.00

“#” = number of isolates; “%” = percentage. Intermediate is the correct CLSI breakpoint category.

#### Antibiotic penetration of *E. coli* biofilms

Among the 43 *E. coli* isolates, 16 were strong biofilm producers, nine were intermediate, and 18 were weak producers. Levofloxacin reduced viable biofilm-embedded *bacteria* by 85%, enrofloxacin by 45%, and the enrofloxacin-colistin combination by 65% (Table 3).

**Table 3:** Antibiotic penetration of *E. coli* biofilms.

Antibiotic	Initial CFU Count (CFU/ml)	Final CFU Count (CFU/ml)	Reduction in Biofilm Viability (%)	Standard Deviation SD)
Levofloxacin	1.0 × 10^6^	1.5 × 10^5^	85%	± 0.05
Enrofloxacin	1.0 × 10^6^	5.5 × 10^5^	45%	± 0.02
Enrofloxacin-colistin	1.0 × 10^6^	3.5 × 10^5^	65%	± 0.03

### In Vivo Studies

#### Growth performance

Levofloxacin-treated birds achieved a significantly higher final body weight (2505.42 ± 36.38 g) and cumulative BWG (2460.37 ± 12.44 g) compared with enrofloxacin-treated birds (2170.64 ± 42.45 g and 2126.09 ± 14.24 g, respectively) and the enrofloxacin–colistin group (2100.54 ± 32.34 g and 2057.94 ± 13.55 g, respectively) (*P* < 0.05). Performance in the levofloxacin group was comparable to the negative control (2520.18 ± 28.67 g and 2476.08 ± 15.33 g, respectively), whereas the positive control group recorded significantly lower values (1640.25 ± 11.78 g and 1595.35 ± 7.34 g, respectively). The levofloxacin-treated group exhibited a significantly improved cumulative FCR (1.43 ± 0.04), comparable to the negative control (1.40 ± 0.07) and significantly better than enrofloxacin (1.64 ± 0.08), enrofloxacin–colistin (1.59 ± 0.06), and positive control (2.05 ± 0.08) groups (*P* < 0.05) (Table 4). Cumulative feed consumption in the levofloxacin group (3521.89 ± 13.27 g) was comparable to the negative control (3460.99 ± 11.24 g) and enrofloxacin group (3479.39 ± 8.74 g), but significantly higher than the positive control (3270.94 ± 8.25 g) and enrofloxacin–colistin group (3281.40 ± 9.75 g). Mortality was lowest in the levofloxacin group (3.33%), substantially below that of the enrofloxacin group (6.67%) and the positive control (23.33%), with peak differences observed during weeks 4 and 5 of the study.

**Table 4:** Growth performance parameters in broilers experimentally infected with *E. coli* O2 (9.0 × 10⁸ CFU/ml) and treated with levofloxacin, enrofloxacin, or enrofloxacin + colistin for five consecutive days.

Parameters	Week	Groups				
		-ve Control	+ve Control	Levofloxacin	Enrofloxacin	Enrofloxacin + Colistin
		1	2	3	4	5
Body Weight (g)	DOC	44.1 ± 0.80a	44.9 ± 0.73a	45.05 ± 0.63a	44.55 ± 0.54a	42.6 ± 0.65a
Body Weight (g)	1	201.35 ± 5.28a	195.15 ± 5.40a	194.35 ± 4.34a	203.2 ± 4.42a	200.5 ± 3.26a
Body Weight (g)	2	552 ± 10.29a	552.64 ± 10.33a	566.83 ± 7.75a	558 ± 7.04a	559.29 ± 8.85a
Body Weight (g)	3	1110.27 ± 15.46a	845.24 ± 20.74b	1139.63 ± 16.66a	1098.55 ± 14.48a	1100.63 ± 19.45a
Body Weight (g)	4	1720.27 ± 16.81a	1192.18 ± 21.35c	1710.75 ± 25.79a	1520.55 ± 23.02b	1439.63 ± 33.18b
Body Weight (g)	5	2520.18 ± 28.67a	1640.25 ± 11.78c	2505.42 ± 36.38a	2170.64 ± 42.45b	2100.54 ± 32.34b
Cumulative Feed Consumption (g)		3460.99 ± 11.24a	3270.94 ± 8.25b	3521.89 ± 13.27a	3479.39 ± 8.74a	3281.40 ± 9.75b
Cumulative Body weight gain (g)		2476.08 ± 15.33a	1595.35 ± 7.34c	2460.37 ± 12.44a	2126.09 ± 14.24b	2057.94 ± 13.55b
Cumulative FCR		1.40 ± 0.07c	2.05 ± 0.08a	1.43 ± 0.04c	1.64 ± 0.08b	1.59 ± 0.06b

Values are expressed as mean ± SEM. Superscript letters within the same row indicate significant differences (*P* < 0.05). BWG = body weight gain; FCR = feed conversion ratio.

#### Hematological parameters

Levofloxacin-treated birds maintained stable hematological parameters throughout the experimental period. Enrofloxacin-treated birds and the positive control group showed notably elevated total WBC counts on days 21 and 28 (28.58 ± 0.72 and 30.3 ± 1.39 × 10³/µl, respectively). RBC counts, Hb concentration, and PCV values did not differ significantly among treatment groups at any sampling point (Table 5).

**Table 5:** Hematological parameters in broilers experimentally infected with *E. coli* O2 and treated for five consecutive days.

Parameters	Day	Groups				
		-ve control	+ve control	Levofloxacin	Enrofloxacin	Enrofloxacin + Colistin
		1	2	3	4	5
RBC (×10⁶/µl)	21	2.63 ± 0.17 a	2.55 ± 0.19 a	2.58 ± 0.10 a	2.51 ± 0.13 a	2.63 ± 0.11 a
	28	2.33 ± 0.18 a	2.52 ± 0.06 a	2.84 ± 0.09 a	2.77 ± 0.11 a	2.4 ± 0.11 a
	35	2.5 ± 0.08 a	2.67 ± 0.15 a	2.31 ± 0.10 a	2.75 ± 0.17 a	2.61 ± 0.19 a
WBC (×10^3^/µl)	21	20.58 ± 0.68 b	32.18 ± 0.31 a	19.28 ± 0.83 b	28.58 ± 0.72 a	30.52 ± 0.66 a
	28	21.14 ± 0.51 b	36 ± 0.70 a	22.2 ± 0.42 b	30.3 ± 1.39 a	32.53 ± 0.95 a
	35	20.42 ± 0.64 a	26.45 ± 0.74 a	21.58 ± 0.68 a	25.22 ± 0.47 a	24.08 ± 0.49 a
Hb (g/dl)	21	7.28 ± 0.74 a	7.22 ± 0.53 a	7.81 ± 0.71 a	7.55 ± 0.33 a	7.63 ± 0.41 a
	28	9.4 ± 0.25 a	9.82 ± 0.43 a	10.57 ± 0.31 a	9.97 ± 0.37 a	9.84 ± 0.22 a
	35	11.7 ± 0.44 a	9.62 **± 0.55a	10.15 *± 0.31 a	10.25 *± 0.20 a	11.26 ± 0.40 a
PCV (%)	21	22.36 ± 0.99 a	20.79 ± 0.85a	23.102 ± 1.05 a	21.6 ± 0.92 a	23.8 ± 1.1 a
	28	41.6 ± 1.63 a	40.3 ± 1.28 a	42.6 ± 1.65 a	38 ± 1.41 a	41 ± 1.41 a
	35	34.68 ± 1.56 a	36.3 ± 1.33 a	37.3 ± 0.60 a	38 ± 1.57 a	34.66 ± 0.20 a

Values are expressed as mean ± SEM. Different superscript letters within the same row indicate significant differences among groups (*P* < 0.05). RBC = red blood cell count; WBC = white blood cell count; Hb = hemoglobin; PCV = packed cell volume.

#### Biochemical parameters

Levofloxacin-treated birds showed significantly lower AST and ALT activities on days 21, 28, and 35 (AST: 43.6 ± 2.57, 50.9 ± 3.40, 53.59 ± 3.12; ALT: 26.1 ± 1.44, 25.99 ± 1.29, 29.06 ± 1.29) compared with enrofloxacin-treated birds (AST: 75.53 ± 2.61, 81 ± 3.35. 83.49 ± 5.17; ALT: 52.23 ± 1.35, 55.75 ± 0.25 a, 55.96 ± 0.35). Serum uric acid and creatinine were significantly elevated in enrofloxacin-treated birds relative to levofloxacin-treated and control birds (Table 6).

**Table 6:** Biochemical parameters in broilers experimentally infected with *E. coli* O2 and treated for five consecutive days.

Parameters	Day	Groups				
		-ve Control	+ve Control	Levofloxacin	Enrofloxacin	Enrofloxacin + Colistin
		1	2	3	4	5
AST (U/l)	21	43.15 ± 1.99 b	84.12 ± 7.51 a	43.6 ± 2.57 b	75.53 ± 2.61 a	73.88 ± 5.15 a
	28	52.00 ± 0.61 b	83.1 ± 6.43 a	50.9 ± 3.40 b	81 ± 3.35 a	80.9 ± 6.36 a
	35	54.3 ± 0.30 b	85.07 ± 5.38 a	53.59 ± 3.12 b	83.49 ± 5.17 a	78.59 ± 4.34 a
ALT (U/l)	21	22.08 ± 1.37 b	66.79 ± 3.77 a	26.1 ± 1.44 b	52.23 ± 1.35 a	50.54 ± 1.73 a
	28	26.29 ± 1.13 b	68.92 ± 2.16 a	25.99 ± 1.29 b	55.75 ± 0.25 a	56.76 ± 0.18 a
	35	26.74 ± 1.41 b	62.54 ± 3.14 a	29.06 ± 1.29 b	55.96 ± 0.35 a	57.38 ± 0.12 a
Serum Uric Acid (mg/dl)	21	8.15 ± 0.21 b	8.11 ± 0.11 b	8.45 ± 0.56 b	18.23 ± 0.24 a	19.05 ± 0.44 a
	28	9.59 ± 0.08 b	9.85 ± 0.09 b	9.8 ± 0.06 b	19.66 ± 0.05 a	19.86 ± 0.04 a
	35	10.12 ± 0.08 b	10.72 ± 0.17 b	10.43 ± 0.06 b	21.41 ± 0.05 a	20.73 ± 0.12 a
Serum Creatinine (mg/dl)	21	0.22 ± 0.003 b	0.26 ± 0.005 b	0.25 ± 0.007 b	1.21 ± 0.08 a	1.24 ± 0.05 a
	28	0.31 ± 0.007 b	0.33 ± 0.009 b	0.29 ± 0.009 b	1.3 ± 0.08 a	1.33 ± 0.07 a
	35	0.33 ± 0.009 b	0.35 ± 0.007 b	0.32 ± 0.005 b	1.34 ± 0.07 a	1.35 ± 0.06 a

Values are expressed as mean ± SEM. Different superscript letters within the same row indicate significant differences (*P* < 0.05). ALT, alanine aminotransferase; AST, aspartate aminotransferase.

#### Oxidative stress parameters

Levofloxacin-treated birds showed significantly reduced MDA levels (7.20 ± 0.72, 6.85 ± 0.86, and 5.57 ± 0.30 nmol/ml) and elevated SOD activity (68.79 ± 5.58, 85.68 ± 4.24, 57.10 ± 4.41 U/mg protein) and GSH concentrations (4.62 ± 0.13, 4.70 ± 0.06, 5.66 ± 0.10 µmol/g) compared with the enrofloxacin-treated birds (MDA: 9.15 ± 0.29, 6.87 ± 0.69, and 6.31 ± 0.15 nmol/ml; SOD: 45.22 ± 2.16, 49.66 ± 3.34, 45.63 ± 2.11 U/mg protein; GSH: 4.18 ± 0.32, 4.66 ± 0.09, 5.32 ± 0.08 µmol/g) on days 21, 28, and 35 (Table 7).

**Table 7:** Oxidative stress parameters in broilers experimentally infected with *E. coli* O2 and treated for five consecutive days.

Parameters	Day	Groups				
		-ve Control	+ve Control	Levofloxacin	Enrofloxacin	Enrofloxacin + Colistin
		1	2	3	4	5
MDA (nmol/ml)	21	7.82 ± 0.10^b^	9.95 ± 0.32^a^	7.20 ± 0.72^b^	9.15 ± 0.29^a^	9.22 ± 0.37^a^
	28	6.25 ± 0.44^b^	7.50 ± 0.69^a^	6.85 ± 0.86^b^	6.87 ± 0.69^a^	7.10 ± 0.29^a^
	35	5.42 ± 0.10^b^	6.88 ± 0.10^a^	5.57 ± 0.30^b^	6.31 ± 0.15^a^	6.43 ± 0.10^a^
SOD (U/mg protein)	21	80.39 ± 7.33^a^	46.72 ± 2.76^b^	68.79 ± 5.58^ab^	45.22 ± 2.16^b^	52.42 ± 1.76^b^
	28	77.29 ± 8.75^a^	47.78 ± 6.17^b^	85.68 ± 4.24^a^	49.66 ± 3.34^b^	57.81 ± 4.71^b^
	35	53.61 ± 0.02^a^	39.28 ± 1.18^b^	57.10 ± 4.41^a^	45.63 ± 2.11^b^	49.82 ± 2.81^b^
GSH (µmol/g)	21	5.12 ± 0.34^a^	3.88 ± 0.06^c^	4.62 ± 0.13^a^	4.18 ± 0.32^b^	4.10 ± 0.15^b^
	28	5.17 ± 0.27^a^	3.36 ± 0.20^c^	4.70 ± 0.06^a^	4.66 ± 0.09^ab^	4.54 ± 0.28^ab^
	35	5.44 ± 0.19^a^	4.03 ± 0.12^c^	5.66 ± 0.10^a^	5.32 ± 0.08^b^	5.27 ± 0.11^b^

Values are expressed as mean ± SEM. Different superscript letters within the same row indicate significant differences (*P* < 0.05). GSH = reduced glutathione; MDA = malondialdehyde; SOD = superoxide dismutase.

#### Microbiological efficacy

On day 21, bacterial recovery from different organs was markedly lower in the Levofloxacin group than in all other infected groups (Table 8).

**Table 8:** Incidence of *E. coli* recovery from visceral organs of broilers experimentally infected with APEC O2 (9.0 × 10⁸ CFU/ml) on day 14, following five days of treatment.

Group	Bone Marrow		Liver		Kidney		Spleen		Lungs	
	#	%	#	%	#	%	#	%	#	%
-ve Control	0	0	0	0	0	0	0	0	0	0
+ve Control	4	80	5	100	4	80	5	100	3	60
Levofloxacin	1	20	1	20	1	20	1	20	1	20
Enrofloxacin	2	40	3	60	2	40	3	60	2	40
Enrofloxacin+ Colistin	2	40	2	40	2	40	2	40	1	20

“#’’ = number of positive samples out of five; “%” = percentage of positive samples. Samples collected on day 21 (*n* = 5 per group).

## Discussion

This study evaluated the comparative therapeutic effects of levofloxacin and enrofloxacin in broilers experimentally infected with APEC with particular attention to growth performance, mortality, hematological and biochemical profiles, oxidative stress responses, and organ-level bacterial clearance. The in vitro susceptibility data underscore the superior antibacterial potency of levofloxacin against biofilm-forming E. coli isolates. Levofloxacin achieved a sensitivity rate of 43.75% among strong biofilm producers, nearly twice that of enrofloxacin (18.75%), and produced significantly larger inhibition zones (26 mm vs. 13 mm), reinforcing its enhanced in vitro efficacy. The higher resistance observed against enrofloxacin (50.00%) compared with levofloxacin (37.50%) is consistent with the well-documented emergence of resistance to second-generation fluoroquinolones in poultry environments^25^. These results support the pharmacological rationale for levofloxacin as an alternative therapeutic agent in biofilm-forming APEC infections^26^. The demonstrated 85% reduction in biofilm-embedded *viable bacteria* by levofloxacin compared with 45% for enrofloxacin and 65% for the enrofloxacin-colistin combination aligns with the findings of Ishida et al.^27^, who documented superior biofilm-penetrating activity of levofloxacin against Pseudomonas aeruginosa.

Levofloxacin-treated birds recorded significantly higher BWG and efficient FCR relative to both the enrofloxacin and enrofloxacin-colistin groups, consistent with prior findings linking levofloxacin therapy to improved nutrient utilization and growth outcomes^28^. These effects likely reflect the drug’s favorable pharmacokinetic properties, including enhanced tissue penetration and a prolonged half-life, which sustain bactericidal concentrations at infection sites and accelerate resolution of systemic disease^28^. The substantially lower mortality in the levofloxacin group (3.33%) compared with enrofloxacin (6.67%) and the untreated positive control (23.33%) further corroborates the superior clinical efficacy of levofloxacin, particularly as disease severity peaks in later weeks of production^29^.

Hematological examination revealed stable erythrocyte parameters across all treatment groups, indicating that neither levofloxacin nor enrofloxacin induced clinically relevant anemia or hematopoietic suppression at therapeutic doses. In contrast, the enrofloxacin-treated and positive control groups displayed marked leukocytosis on days 21 and 28, reflecting persistent systemic inflammatory activity consistent with septicemic colibacillosis^30,31^. The comparatively normalized WBC counts in levofloxacin-treated birds suggest more complete bacterial clearance and attenuation of systemic inflammation, as noted by Varia et al.^32^ for third-generation fluoroquinolones. Mostafa^29^ reported transient, mild reductions in RBC and Hb on days 5–7 post-levofloxacin treatment that resolved by day 9, underscoring the importance of adhering to recommended dosage regimens.

Levofloxacin-treated birds exhibited significantly lower ALT and AST activities across all sampling periods compared with enrofloxacin-treated birds, indicating reduced hepatocellular injury and better hepatic integrity—consistent with findings reported by Varia et al.^32^. Furthermore, serum uric acid and creatinine concentrations were markedly elevated in enrofloxacin-treated groups, pointing to greater renal metabolic stress or nephrotoxic risk under the conditions of the experimental challenge^33^. The relatively stable renal biomarkers in the levofloxacin group suggest superior renal tolerance, in agreement with Varia et al.^32^. Discrepancies between studies in which minimal biochemical differences between fluoroquinolones were observed may reflect differences in dosage regimens, infection severity, and exposure duration, emphasizing the importance of contextual interpretation of fluoroquinolone safety data.

Oxidative stress constitutes a critical pathological axis in APEC infection, driving tissue damage and exacerbating disease severity^34^. In the present study, levofloxacin treatment markedly reduced MDA concentrations and restored SOD activity and GSH concentrations toward values comparable to the negative control, suggesting effective mitigation of oxidative damage—most likely through superior bacterial clearance and reduction in endotoxin-mediated reactive oxygen species (ROS) production. Similar antioxidant benefits of levofloxacin have been reported by Al-Safi and Kafi^35^ and Mostafa^29^. In contrast, enrofloxacin-treated birds showed only partial normalization of oxidative stress markers, possibly reflecting slower infection resolution or fluoroquinolone-associated mitochondrial ROS generation^36,37^. The enrofloxacin–colistin combination conferred no additional antioxidant benefit and, in some instances, showed oxidative profiles comparable to or exceeding those of enrofloxacin alone, potentially attributable to the known oxidative and nephrotoxic effects of colistin. Under infectious challenge, these findings indicate that levofloxacin provides superior redox protection compared with enrofloxacin, an advantage that supports tissue integrity and recovery in commercial broiler production.

The markedly lower APEC recovery rates from organs in levofloxacin-treated birds on day 21 further corroborate its superior microbiological efficacy, consistent with findings of near-complete bactericidal activity at minimum inhibitory concentration (MIC) levels within 6 h^26^ and with the broad sensitivity of APEC and related pathogens to levofloxacin^38^.

## Conclusion

The findings of this study indicate that levofloxacin is more effective than enrofloxacin for the treatment of avian colibacillosis in broiler chickens. Levofloxacin treatment achieved greater cumulative body weight gain, more favorable FCR, reduced mortality, and improved hematological, biochemical, and antioxidant profiles compared with enrofloxacin. Superior microbiological efficacy, as evidenced by greater biofilm penetration and lower bacterial recovery from organs, further supports levofloxacin’s role as a viable and therapeutically advantaged alternative for managing APEC infections, with potential benefits for animal welfare and commercial productivity.

## Data Availability

The data sets spent and/or analyzed during this study are available from the corresponding author upon reason able request.
